# Confinement and the Hatred of Sound in Times of COVID-19: A Molotov Cocktail for People With Misophonia

**DOI:** 10.3389/fpsyt.2021.627044

**Published:** 2021-05-10

**Authors:** Antonia Ferrer-Torres, Lydia Giménez-Llort

**Affiliations:** ^1^L'Alfatier - Centro Médico Psicológico, Barcelona, Spain; ^2^Department of Psychiatry and Forensic Medicine, School of Medicine, Universitat Autònoma de Barcelona, Barcelona, Spain; ^3^Institut de Neurociències, Universitat Autònoma de Barcelona, Barcelona, Spain

**Keywords:** confinement, COVID-19, Coronasomnia, psychologic symptoms, psychosomatic symptoms, sleep disorders, secondary impact, gender medicine

## Abstract

Forced strict confinement to hamper the COVID-19 pandemic seriously affected people suffering from misophonia (M+) and those living with them. Misophonia is a complex neurophysiological and behavioral disorder of multifactorial origin, characterized by an intense physiological and emotional response produced by intolerance to auditory stimuli of the same pattern, regardless of physical properties. The present work studied the secondary impact that strict confinement caused in 342 adults (224 women: 118 men) regularly attending a medical psychological center in Barcelona. Misophonia, usually underdiagnosed, showed a prevalence of 35%, the same for women (37%) than men (31%). A retrospective analysis using a physical-psychological-social inventory of 10 variables evaluated the number of individuals that during confinement and self-confinement (March 11 - June 29, 2020) canceled (mostly M-) and/or requested a therapeutic intervention, the reasons for their request, and the strategies they used to self-manage the situation. Ten main variables indicated that the confinement exponentially increased the effects of misophonia compared with results from the same individuals during the last quarter of 2019. Most people diagnosed with misophonia continued with tele-assistance during the confinement because of this impact's self-concern. Besides the impacts as part of the general population, M+ also developed different symptoms causing significant personal, social, and job/occupational imbalance, as compared to M-. Health, fears, conflicts with neighbors, study-related difficulties were outstanding reasons for consultations. The LSB-50 test for ‘Psychological and Psychosomatic Symptoms’ applied to M+ revealed the increase of 8 of 9 items of this psychopathological test. Sleep disorders (coronasomnia), hostility, depression, and somatization were more severe than in previous assessments. Women presented the worst psychological and psychosomatic states (eight out of nine, as compared to one out of nine in males). The study unveiled the complex physical-psychological-social burden, the need for dissemination and a gender perspective to understand the secondary impact of COVID-19 pandemic on the mental health of the population with misophonia. The results also show that in this new COVID era people suffering from misophonia need to develop coping strategies addressing modifiable risk and protective factors. They deserve familial/social comprehension, stronger clinical support and a gender medicine perspective.

## Introduction

On March 11, 2020, the WHO declared the global pandemic by COVID-19 (1). In Spain, 6 days later, an unprecedented period of strict confinement of the entire 47 million population began as part of the “declaration of the state of alarm for managing the health crisis caused by COVID-19”. Subsequently, other countries in Europe and other continents adopted similar measures in the face of the evident advance of the pandemic and tragedy left behind (2, 3). One year later, confinement still is an option to hamper the spread of the virus even though severe confinement measures, quarantine, and social isolation exert a significant economic, social, and psychological impact (4). At the psychological level, the feelings of frustration, restlessness, irritability, hostility, uncertainty, sadness, fear, and anger are just a sample of the long list of negative emotions that people in a situation of “forced confinement” describe (5–7). Besides, the domestic space's reorganization, shared daily life, home multitasking, and intrusion into intimate life through virtual systems are among many other stressors imposed by the current sanitary crisis. The emergency and uncertainty on the coronavirus outbreak have not allowed the necessary physical or emotional adaptation in a rapidly changing scenario, which results in highly stressful situations in the individuals and society (8).

Epidemics are known to increase psychiatric morbidity and exert remarkable emotional distress (9). Besides the distress induced by the COVID-19 pandemic, the people with misophonia - a quite unknown, underdiagnosed, and untreated neurophysiological disorder, also referred to as “the hatred of sounds” (10)- confronted the constant and inescapable exposure to unwanted/unpleasant sounds [for the definition of noise please refer to the review by Erfanian et al. (11)] potentially disturbing for them. This complex neurophysiological and behavioral disorder is characterized by increased physiological responsiveness and a high degree of emotional reactivity due to intolerance to specific auditory stimuli, as described by (12). Since then, research of misophonia have considerably evolved (10, 13–17)]. People with misophonia experience intense physical, behavioral, and emotional “misophonic responses” when exposed to the so-called “misophonic sounds” or “aversive triggers” that are part of every day's sound, but they differ from one individual to another (14, 18, 19). The most disturbing sounds are mouth-related sounds followed by nose-related ones (20), but other humans, animals, objects, and environmental sounds are part of an extensive list of triggers. People with misophonia may also experience an aversion to stimuli in movement known as misokinesia (13) or “visual triggers” (15, 16).

As with any other pathology or disorder, the therapeutic or social protection that could allow the individuals with misophonia to cope with the confinement was scarce. The stay-home restriction was a potential Molotov cocktail. As reported by the news (21–25), the different Spanish autonomous communities were forced to reinforce citizen security measures, launch extra mediation services, and promote campaigns for coexistence to alleviate neighborhood conflicts that occurred during confinement. According to the same sources, intra-family conflicts were expressed more frequently and forcefully in this stressful situation.

This study aimed to examine a secondary impact of COVID-19, the physical-psycho-social effects that the exceptional situation of confinement during March and April 2020 generated in a sample of patients regularly attending a medical psychology center in Barcelona, among them, people diagnosed with misophonia. Most misophonic individuals exhibited a “self-confinement” behavior once the mandatory reclusion was over because of their fear of getting infected, ill, or dying of COVID-19. Therefore, the study was extended until the end (29th) of June 2020 to consider these aspects. Four specific aims were defined to know (1) the changes in the individuals' behavior concerning the request for help or consultation and the therapeutic intervention they were carrying out. (2) the reason for the request or consultation regarding the therapeutic intervention, (3) if they could self-manage the situation reason for consultation or request for help, and (4) the identification and assessment, using the LSB-50 scale, of 'Psychological and Psychosomatic Symptoms' in adults diagnosed with misophonia, and the interference in the individual's personal and social functioning.

## Materials and Methods

### Subjects

This study was carried out with a total sample of 342 people regularly attending a Medical and Psychological Center in the city of Barcelona (Spain). The data were analyzed in a double-blind manner to eliminate the confirmation bias.

Inclusion criteria were defined as follows: Women and men over 16 years of age that signed (legal tutors, in the case of minors) the informed consent; diagnostic test battery before August 2019; agreement to participate in the study. In the second phase of the study with patients with misophonia: at least mouth-related and nose-related trigger sounds; self-confinement, leaving home only to carry out emergency or essential situations.

Exclusion criteria were defined as follows: Adults or minors whose parents or guardians did not sign the informed consent or refused to participate in the study; those under 16 years of age; blind people; people with profound deafness. M+ without mouth-related and nose-related sounds as triggering stimuli. During the study, those people who during confinement and self-confinement carried out social gatherings or leisure activities outside the family home were discarded.

### Evaluation Tools

#### Record of Request and Reason for Consultation (2RC, Phase I)

The 2RC is a self-report questionnaire developed by the medical psychology center used as a historical record that begins with the first contact and ends with discharge. All the individuals' requests, comments, and consultations are also recorded, regardless of the therapeutic work. Four categories are distinguished: (1) registration of visits, cancellations, and changes in programming; (2) physical health; (3) psychological health; (4) social well-being: relationships (family, friends, and neighbors), work, economy, and studies.

#### Misophonia Scale

The participants were classified as diagnosed with misophonia (M+ > = 5) or not (M- < = 4), according to the A-MISO-S = Amsterdam misophonia scale (13).

#### Psychological and Psychosomatic Symptoms (PPS, Phase II)

In the sample of participants with a positive diagnosis of misophonia (M+), a psychopathological evaluation tool, the *LSB-50 Brief Symptom List* (26), was used for a pre-post evaluation of the symptoms the test refers as PPS. This tool contains nine clinical scales and subscales, as follows: Psychoreactivity, Hypersensitivity, Obsessive-compulsive, Anxiety, Hostility, Somatization, Depression, Sleep disturbances (Sleep disorder and Amplified sleep disorder).

### Procedure

#### Phase I – Request and Reason for Consultation (2RC) in People Attending the Center

**Figure 1:**
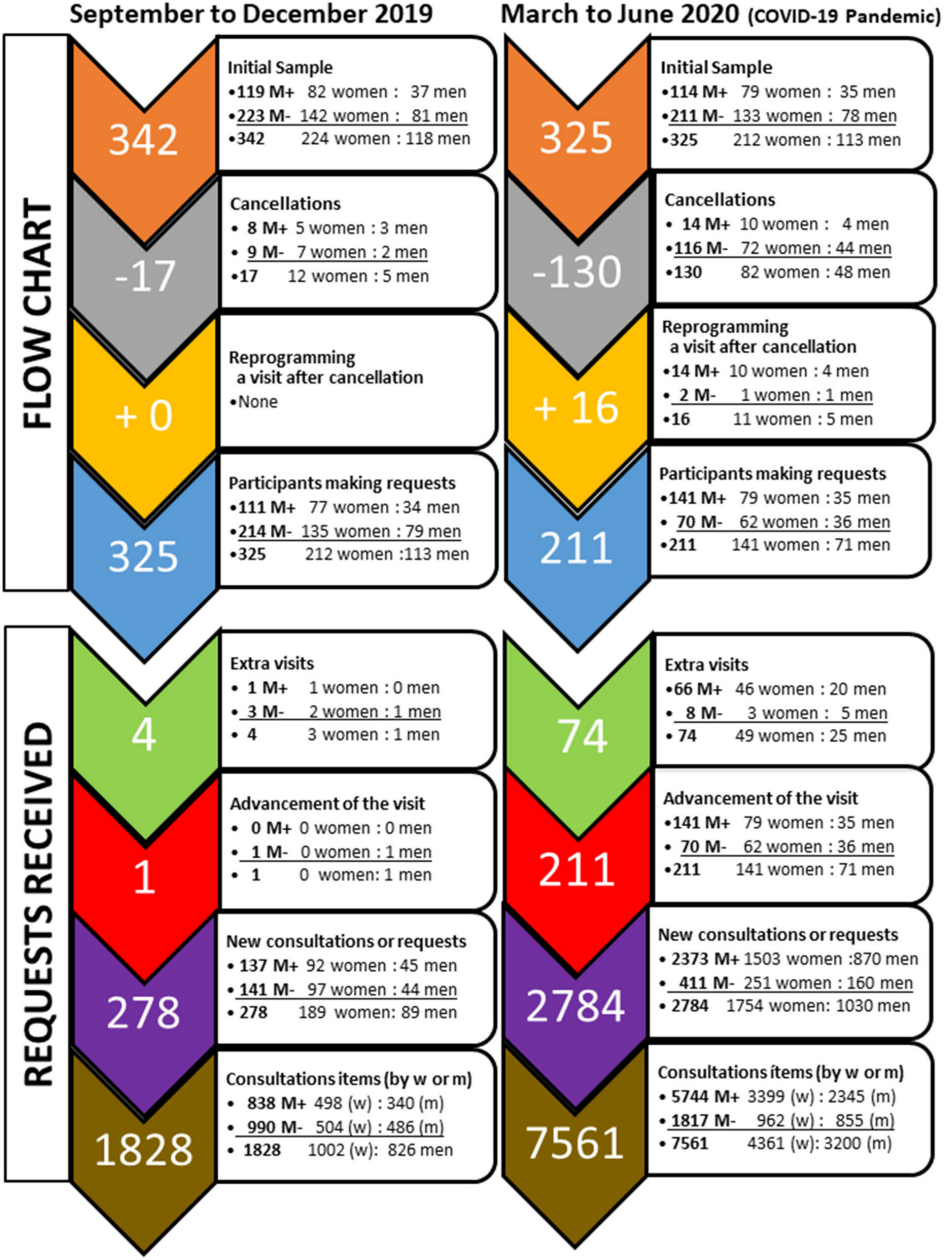
Flow chart of participants in the two periods studied and number of requests received in the medical psychology center.

A retrospective analysis of 2RC of the participants was carried out to compare the quarter of the pandemic (March 11 to June 29, 2020) with the last quarter of 2019 (September to December), used as a reference (see Figure 1). The criteria by which this reference quarter was chosen were: (a) the data from the 2RC during this quarter was an average reference for the quarters analyzed during the years 2017 to 2019; (b) since both quarters were temporally close, there would have been few registrations, and therefore the participants in both studied periods would be the same and, (c) it was ruled out to compare them with January and February 2020 because together with July and August, they are periods where the number of individuals attending the center is irregular. On March 11, 2020, all individuals were informed that, as a preventive measure to stop the coronavirus pandemic spread, all group activities were canceled, but the face-to-face visits would remain open for those who need to maintain the quarterly schedule. This support was provided through telephone, videoconferences, e-mails, and WhatsApps.

From the 2RC, the number of participants, frequency, and reasons for requests was extracted as follows:

1. *Number of participants and frequency of requests, before and after the confinement:* The sample of participants, the number of individuals who canceled their visits, and those that requested new programming after a cancellation were recorded for each period. The advancement of a visit and the extra visits were also recorded. Regardless of attendance at scheduled visits, the individuals could request a series of extra resources. New consultations contacting the center for one or more reasons and requests of visits were noted. 'Consultation items' refers to the number of items, topics, problems, or aspects that the individuals requested.
2. *Reason for requests or inquiries*. The 2RC recorded concerns, problems, doubts, fears, and other aspects of the request for help or an extra resource. Analysis of requests was done according to the physical-psychological-social dimensions and categories (see Tables 1, 2). The data were also analyzed in a segregated manner, according to sex and misophonia diagnosis (as depicted in Figure 1).

**Table 1:** Results are expressed as total number and, in parenthesis, as the percentages.

	**September to December 2019**			**March to June 2020**			**Fold increase and Statistics**		
	**Total (%)**	**Women (%W)**	**Men (%M)**	**Total (%)**	**Women (%W)**	**Men (%M)**	**Total**	**Women**	**Men**
**Participants**									
Sample of participants (*n*, %)	342 (100)	224 (65)	118 (35)	325 (100)	212 (65)	113 (35)	1.0	0.9	1.0
M+: M-	119:223	82:142	37:81	114:211	79:133	35:78	1:1		
(%M+: %M-)	(35:65)	(37:63)	(31:69)	(35:65)	(37:63)	(31:69)	*n.s*.		
Cancellation (*n*, %)	17 (5)	12 (4)	5 (1)	130 (40)	82 (63)	48 (37)	7.6^***^	6.8^***^	9.6^***^
M+: M-	8:9	5:7	3:2	14:116	10:72	4:44	1.8:13		
(%M+: %M-)	(47:53)	(42:58)	(60:40)	(11:89)	(12:88)	(8:92)	M, *p* < 0.001		
Reprogramming (*n*, %)	0 (0)	0 (0)	0 (0)	16 (5)	11 (69)	5 (31)	16.0^***^	11.0^***^	5.0^*^
M+: M-	0:0	0:0	0:0	14:2	10:1	4:1	14:2		
(%M+: %M-)	(0)	(0:0)	(0:0)	(88:12)	(91:9)	(80:20)	M, *p* < 0.001		
Making requests (*n*, %)	325 (95)	212 (65)	113 (35)	211 (65)	141 (67)	70 (33)	0.6^***^	0.7^***^	0.6^***^
M+: M-	111:214	82:130	37:76	114:98	79:62	35:35	1:0.5		
(%M+: %M-)	(34:66)	(39:61)	(33:67)	(54:46)	(56:44)	(50:50)	M, *p* < 0.001		
Extra visits (*n*, %)	4 (1)	3 (75)	1 (25)	74 (35)	49 (66)	25 (34)	18.5^***^	3.0^***^	25.0^***^
M+: M-	1:3	1:2	0:1	66:8	46:3	20:5	66:2.7		
(%M+: %M-)	(25:75)	(33:67)	(0:100)	(89:11)	(94:6)	(80:20)	M, *p* < 0.001		
Advancing the visit (*n*, %)	1 (0.31)	0 (0)	1 (100)	10 (5)	6 (60)	4 (40)	10.0^***^	6.0;^**^	4.0 *n.s*.
M+: M-	0:1	0:0	0:1	6:4	4.2	2:2	6:4		
(%M+: %M-)	(0:100)	(0:0)	(0:100)	(60:40)	(64:36)	(50:50)	M, *p* < 0.01		
**Consultations**									
New consultations (*n*, %)	278 (100)	189 (68)	89 (32)	2,784 (100)	1,754 (63)	1,030 (37)	10.0 n.a.	9.3 n.a.	11.6 n.a.
M+: M-	137:141	92:97	45:44	2,373:411	1,503:251	870:160	140:2.9		
(%M+: %M-)	(49:51)	(49:51)	(51:49)	(85:15)	(86:14)	(84:16)	M, *p* < 0.001		
Consultation items (*n*, %)	1,828 (100)	1,002 (55)	826 (45)	7,561 (100)	4,361 (58)	3,200 (42)	4.1 n.a.	4.4^*^	3.9^*^
M+: M-	838:990	498:504	340:486	5,744:1817	3,399:962	2,345:855	7:1.8		
(%M+: %M-)	(46:54)	(50:50)	(41:59)	(76:24)	(78:22)	(73:27)	M, *p* < 0.001		

*In the totals, percentages are calculated vs. the initial sample. (0), <1%. In women and men, (%W) and (%M) percentages represent the women:men ratio. Statistics: Chi-square with Yates' correction or Fisher's exact test; n.a., not applicable; n.s. not statistically significant*

* *p < 0.05*;

** *p < 0.01*;

*** *p < 0.001*,

*relative increase vs. the global increase observed in the “March to June 2020” period. Factor M, Misophonia; p < 0.01; p < 0.001 as compared to fold increase in the M- counterpart. The significance of the bold values is that they are the values that have presented a higher increment from the first phase to the final phase*.

**Table 2:** Results are expressed as total number and (percentages).

		**September to December 2019**			**March to June 2020**			**Fold increase & Statistics**		
**Dimension**	**Level**	**Total (%)**	**Women (%W)**	**Men (%M)**	**Total (%)**	**Women(%W)**	**Men (%M)**	**Total**	**Women**	**Men**
Global	Global	1,828 (100)	1,002 (55)	826 (45)	7,561 (100)	4,361 (58)	3,200 (42)	4.1 n.a.	4.4^*^	3.9^*^
Physical	Physical health	182 (10)	105 (58)	77 (42)	406 (5)	231 (57)	175 (43)	2.2^***^	2.2^***^	2.3^***^
	M+: M-	90:92	57:48	33:44	339:67	184:47	155:20	3.8: 0.7		
	(%M+: %M-)	(49:51)	(54:46)	(43:57)	(84:16)	(80:20)	(89:11)	M, *p* < 0.001		
Psychological	Psychology	1,272 (70)	676 (53)	596 (47)	5,544 (73)	3,193 (58)	2,351 (42)	4.4^**^	4.7^***^	3.9
	M+: M-	667:605	350:326	317:279	5,137:407	2,987:206	2,150:201	7.7:0.7		
	(%M+: %M-)	(52:48)	(52:48)	(53:47)	(93:7)	(93:6)	(91:9)	M, *p* < 0.001	S, *p* < 0.01	
Social	Total Social	374 (21)	221 (60)	153 (40)	1,611 (20)	937 (58)	674 (42)	4.3	4.2	4.4
	M+: M-	199:175	126:96	74:80	1,310:301	787:151	564:111	6.6: 1.7		
	(%M+: %M-)	(53:47)	(57:43)	(48:52)	(81:19)	(84:16)	(84:16)	M, *p* < 0.001		
	Family	121 (7)	77 (64)	44 (36)	473 (6)	275 (58)	198 (42)	3.9	3.6	4.5
	M+: M-	66:55	45:32	21:23	358:115	221:54	177:21	5.4: 2.1		
	(%M+: %M-)	(55:45)	(58:42)	(48:52)	(76:24)	(80:20)	(89:11)	M, *p* < 0.001		
	Friends	19 (1)	12 (63)	7 (37)	26 (0)	16 (62)	10 (38)	1.4^***^	1.3^**^	1.4
	M+: M-	9:10	5:7	4:3	13:13	9:7	4:6	1.4: 1.3		
	(%M+: %M-)	(47:53)	(42:58)	(57:43)	(50:50)	(56:44)	(40:60)	*n.s*.		
	Neighbors	49 (3)	29 (59)	20 (41)	413 (5)	248 (60)	165 (40)	8.4^***^	8.6^***^	8.3^**^
	M+: M-	34:15	20:9	14:6	361: 52	224:24	137:28	10.6: 3.4		
	(%M+: %M-)	(69:31)	(69:31)	(70:30)	(87:13)	(90:10)	(83:17)	M, *p* < 0.01		
	Work	91 (5)	49 (54)	42 (46)	317 (4)	182 (57)	135 (43)	3.5	3.7	3.2
	M+: M-	38:53	23:26	15:27	282:35	162:20	120:15	7.2: 0.7		
	(%M+: %M-)	(42:58)	(47:53)	(36:64)	(89:11)	(89:11)	(89:11)	M, *p* < 0.001		
	Economy	62 (3)	34 (55)	28 (45)	201 (3)	116 (58)	85 (42)	3.2	3.4	3.0
	M+: M-	32:30	20:14	12:16	133:68	80:36	53:32	4.1: 2.6		
	(%M+: %M-)	(52:48)	(59:41)	(43:57)	(66:34)	(69:31)	(62:38)	M, *p* = 0.0546		
	Studies	30 (2)	19 (63)	11 (37)	179 (2)	99 (55)	80 (45)	6.0	5.2	7.3
	M+: M-	19:11	12:7	7:4	162:17	90:9	72:8	8.5: 1.5		
	(%M+: %M-)	(63:37)	(63:37)	(64:36)	(90:10)	(91:9)	(90:10)	M, *p* < 0.001		
	Other	2 (0)	1 (50)	1 (50)	2 (0)	1 (50)	1 (50)	1.0	1.0	1.0
	M+: M-	1:1	1:1	1:1	1:1	1:1	1:1	1:1		
	(%M+: %M-)	(50:50)	(50:50)	(50:50)	(50:50)	(50:50)	(50:50)	*n.s*.		

*In the totals, percentages are calculated vs. the initial sample. (0), <1%. In women and men, (%W) and (%M) percentages represent the women:men ratio. Statistics: Chi-square with Yates' correction or Fisher's exact test; n.a., not applicable; n.s. not statistically significant*

* *p < 0.05*;

** *p < 0.01*;

*** *p < 0.001*,

*relative increase vs. the global increase observed in the “March to June 2020” period. Factors: M, Misophonia; S, sex; p < 0.01; p < 0.001 as compared to fold increase in the counterpart*.

#### Phase II – Psychological and Psychosomatic Symptoms (PPS, LSB-50) in People With Misophonia

The second phase of the study focused the investigation on the participants with a positive diagnosis of misophonia. Once the mandatory confinement finished, during self-confinement, the LSB-50 was administered to participants in person from June 2 to 29, 2020. The results were compared with those obtained during the patient's diagnostic evaluation, which was carried out between 6 and 9 months before the quarter of the pandemic. The nine clinical scales and subscales of LSB-50 were taken into account. Changes in the patients' “Psychological and Psychosomatic Symptoms” (PPS) during the pandemic quarter were calculated through the percentage difference to the quarter of reference (September to December 2019). The LSB-50 questionnaire was corrected using a computerized system to eliminate any confirmation bias from the first part of the study.

### Statistical Analysis

SPSS 20.0 software was used. Continuous variables are expressed as mean ± SD, percentage, or percentile. Sex and Misophonia ratios are shown in percentage as W: M, woman:men, M+:M- patients with misophonia: patients without misophonia. The chi-square with Yates' correction, or when necessary Fisher's exact test, was used for between-groups comparisons. A *p* < 0.05 was considered statistically significant.

## Results

### Demographic data

The sample of 342 participants had a 65:35 W: M ratio and a mean age of 44.66 ± 14.09 years (min 16, max 89 years). Half of them were married or cohabiting (51%), 25% were single, a similar percentage were divorced (23%), while 2% were widows (all of them, women).

### Phase I – Request and Reason for Consultation (2RC) in People Attending the Medical Psychology Center

#### Number of Participants and Frequency of Requests

The flow chart of participants and frequency of requests during the two periods of time are summarized in Figure 1. The records of 2RC for the two studied quarters are described in Table 1. First, the data for the sample and each sex are given. The data are further depicted to detect distinct behaviors between participants according to a positive (M+, prevalence of 35%) or negative (M-) diagnosis of misophonia.

During the reference quarter, the standard behavior of individuals concerning attendance to the center was characterized as a low cancelation ratio, it was independent of the diagnosis of misophonia, and no one requested an extra visit. A few other people requested to increase the number of visits (half of them were M+) or advance them. The standard number of consultations was 278 to approach a total of 1828 items.

During the pandemic, cancellations increased compared to the quarter of reference. Women and men exhibited similar cancellation ratios. However, the cancellations were mainly associated with M-, while the cancellation ratio in M+ was not modified. The patients' behavior regarding reprogramming a visit after cancellation, extra visits, and advancement of visits was also modified. Most people preferred to postpone their visit once the risk of contagion had passed. Very few referred or justified the requests due to unforeseen events and variations in their agenda. A few participants requested a reprogramming after the cancellation, a behavior not observed in the previous quarter. In contrast, most of M+ maintained their schedules through telematic service despite the confinement. For these reasons, the 16-fold increase in visits was mainly associated with M+ people.

Concerning the number of participants making requests, 95% of them (M+ and M-) did so before the pandemic. Since the number of cancellations from March to June was high among the M- individuals, the number of them that finally made a request was reduced to half.

In the sample of 211 participants who made a request during the pandemic (see Figure 1, bottom and Table 1), the number of extra visits and advanced visits increased compared to the quarter of reference. The number of extra visits exhibited an 18.5-fold increase. This represented a 2.7-fold increase in M- group and a 66-fold increase in M+. Requests for extra visits were similar for M+ women and men, at a rate of approximately 0.6 extra visits per person. This was significantly higher than the rate of requests for extra visits by M- men and women, at a rate of 0.04 extra visits per person. Advancement of the visit, also scarce during the quarter of reference, increased 10-fold during the pandemic, as it happened for the first time in M+ people. In the case of M-, statistics yield their 4-fold increase as not reaching statistical significance.

Overload of demands in the pandemic resulted in a 10-fold increase in new requests. Also, there was a 4.1-fold increase in the consultation items. New consultations per person were 10 times greater for M+ than M- (20.8 per M+ patient, 1.9 per M- patient). The increase of new consultations for M+ translated into an increase in the consultation items.

#### Reason for Requests or Inquiries

As detailed in Table 2, in the quarter of reference, the most frequent topic for requests or inquiries was referred to psychology. In a lower number, requests referred to social and physical aspects. In the social context, the family and work questions were more frequent, while a minor part referred to neighbors and the economy. Finally, a few were related to studies and friends.

During the pandemic, the total number (global) consultation items exhibited a 4.1-fold increase. Significant differences with the quarter of reference were found as an increase in the number of concerns about psychology, that in women raised to a 4.7-fold increase, significantly higher than the 3.9 increase observed in men. The psychological burden was significantly increased in M+ patients as compared to M-. At the social level, most consultations referred to neighbors. The total amount of concerns about friends and physical health were smaller but still significant. The increases were higher in patients diagnosed with misophonia than M- in the total social items, family issues, work and studies, and neighbors.

In both quarters, the fear that worried the patients the most was related to hypervigilance to noise and/or movement. In both cases, this fear was formulated by people diagnosed with misophonia. The requests referred to a state of expectation and greater sensory sensitivity to the experience of living (during confinement and self-confinement), a higher amount of auditory and visual stimuli, both related to neighbors and the family. The aversive sounds reported were related to the intensified and overlapped activities (walking, homework, playing, tv, keyboard, singing) of their own family or neighbors. They also included new social expressions of support (songs, hand-clapping, music, etc.) from neighbors standing in the balconies as they irrupted the silenced cities. According to clinical interviews, this hypervigilance and hypersensitivity affected other aspects of health, neighborhood, and family life.

#### Phase II – Psychological and Psychosomatic Symptoms (PPS) in People With Misophonia

Concerning the prevalence of misophonia, the diagnosis was positive in 114 of the 325 participants (35% of the patients) and was similar for women (37%) than for men (31%). Interestingly, only 2% of them initially went to the center to be treated for this problem since most of them (98%) were admitted due to other psychological or psychosomatic problems and were unaware of misophonia. In this second part of the study, these 114 M+ were studied to determine the pandemic's secondary impact on psychological and psychosomatic symptoms.

In the interviews conducted during the LSB-50 assessment, most of the patients diagnosed with misophonia, women and men, self-reported a progressive increase in symptoms during the pandemic quarter. These results were in agreement with the patient records in the first part of the study.

**Figure 2:**
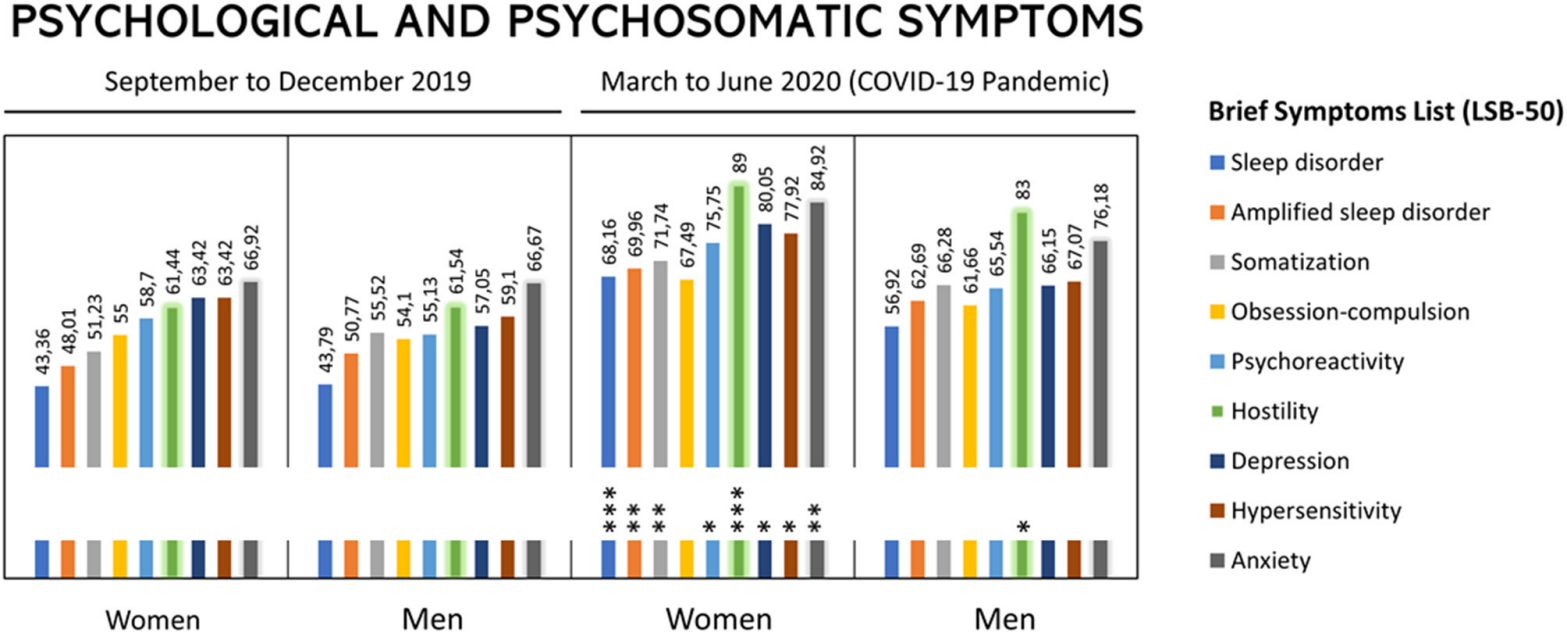
Changes in the Psychological and Psychosomatic Symptoms during the confinement (Barcelona, March to June, 2020) due to COVID-19 pandemic in with women and men with a positive diagnosis of misophonia. Results are expressed as percentiles. Brief symptoms list (LBS-50) as provided in the legend. Asterisks refer to symptoms that increased dramatically in the second period of the study.

As illustrated in Figure 2 and depicted in Table 3, in the reference period, the nine 'Psychological and Psychiatric Symptoms' assessed with LSB-50 showed similar results for women and men, with the lowest percentiles for “sleep disorder” and the maximum percentiles in “anxiety”. Interestingly, during the pandemic quarter, 8 of the nine symptoms were found significantly increased in women, 1 of the nine in men. The symptoms showed different degrees of severity and gender bias since they were worse in women. In particular, “hostility” reached the higher percentiles in both sexes, followed by “anxiety.” “Sleep disorders” and “amplified sleep disorders” showed the highest increases, mainly in women. Percentiles of psychoreactivity and hypersensitivity were increased in women, while men did so in a lower percentage.

**Table 3:** Phase II - Psychological and psychosomatic symptoms (PPS) in people with misophonia.

**Brief Symptom List**	**September to December 2019**		**March to June 2020**		**Increase**	
**(LSB-50)**	**Women**	**Men**	**Women**	**Men**	**Women (%)**	**Men (%)**
Psychoreactivity	58.7	55.1	75.8^*^	65.5	17.1 (29)	10.4 (19)
Hipersensitivity	63.4	59.1	77.9^*^	67.1	**14.5 (23)**	**8.0 (14)**
Obsession-compulsion	55.0	54.1	67.5	61.7	**12.5 (23)**	**7.6 (14)**
Anxiety	**66.9**	**66.7**	84.9^**^	76.2	**18.0 (27)**	**8.7 (14)**
Hostility	61.4	61.5	89.0*	83.0*	**27.6 (45)**	21.5 (35)
Somatization	51.2	55.5	71.7^**^	66.3	**20.5 (40)**	**10.8 (16)**
Depression	63.4	57.1	80.1^*^	66.2	16.7 (26)	9.1 (16)
Sleep disorder	43.4	43.8	68.2^***^	56.9	**24.8 (57)**	**13.1 (30)**
Amplified sleep disorder	48.0	50.8	70.0^**^	62.7	**22.0 (46)**	**11.9 (23)**

*Results are expressed as total percentiles or increase in units or (percentage) segregated for women and men with misophonia. Statistics: Chi-square with Yates' correction*;

* *p < 0.5*;

** *p < 0.01*;

*** *p < 0.001 vs. the period before the pandemic*.

*The significance of the bold values is that they are the values that have presented a higher increment from the first phase to the final phase*.

## Discussion

This research studied the secondary impact of the COVID-19 pandemic on people attending a medical psychological center in the city of Barcelona during the first quarter March-June, 2020. We analyzed the frequency and reasons for requests for help and therapeutic interventions. Among them, their fear of death or the death of others and worries about the multiple loss caused by COVID-19, including work, economy, and studies. The descriptive study compared their behavior with the behavior shown in the quarter of September-December, 2019, chosen as reference quarter. The number of cancellations due to the fear of being infected was high. When the presence of a positive diagnosis of misophonia was analyzed, the results showed that most (96 %) of participants diagnosed with misophonia maintained their visits. Therefore, the second part of the study was aimed to analyze with LSB-50 the impact of confinement and self-confinement in the PPS of this specific sample population, since worsening of hostility, depression, and anxiety can be triggers of difficult situations in such scenarios.

### Variations in the Treatment Schedule in People Attending the Center

The first remarkable observation was the greater number of requests and cancellations than those made during the reference quarter. Half of the patients canceled their quarterly schedule, and most of them justified this decision due to the state of alarm decreed by the government. When the alarm had risen, they argued the fear of being infected and infecting others, resulting in self-confinement behaviors. A small percentage of individuals indicated personal reasons unrelated to the pandemic situation. This behavior has also been described in other clinical scenarios and in many countries [i.e., (27)], where the number of patients regularly attending medical supervision for chronic diseases (i.e., cardiovascular) was dramatically reduced. The potential risks of this behavior contributing to the increase in the pandemic's secondary impact were worrisome, to the extent that medical doctors warn their patients, and the general population was warned about it in the TV news.

The data segmentation for misophonia diagnosis indicated that the arguments provided by M- contrasted with the low number and reasons for cancellations of those diagnosed with misophonia. In advance, misophonic patients feared that there would be a greater amount of misophonic stimuli in the confined situation. Therefore, they estimated that therapeutic help would be needed. The M+ participants were part of a specific program about misophonia, that probably allowed them to contextualize their problem and take the appropriate preventive measures. The results also confirm the relevance of knowledge of the problem and the diagnosis as the first steps to avoid misophonia being an ignored and, therefore, untreated problem.

### Reasons of Consultations in People Attending the Center

The reasons for the requests for help and consultations were analyzed. These kinds of therapeutic requests are those that patients refer to the professional, independently of their clinical visits. Every time the patient contacts the center's professionals to request their support, the consultation or request for help is included in the record of “Requests and Reasons for Consultation” (2RC). Each consultation or request for help can include more than one item or reason for consultation. The results described show that, during the pandemic quarter, requests for extra visits increased 18-fold, in addition to the visits already assigned. This finding reflects the discomfort that patients experienced during the period of confinement and self-confinement. Another data that quantified the increase in psychological interventions was the number of requests and consultation items. During the reference quarter, the medical center received 278 requests for assistance that resulted in a total of 1,828 consultation items. During the confinement, the number of requests increased ten times (2,784 requests) and resulted in 7,561 (314%) consultation items. These increases are in agreement with the predictions and later confirmation of an overload of psychological burden and support demands recorded in the first wave of the pandemic (28, 29), referred to as being also increased (not yet quantified) in the second and current third wave of this pandemic. From a gender perspective, the ratio of requests between women and men was similar in both quarters. However, as quantified in the second part of the study, women had greater increase in severity of misophonia during the pandemic based on the LSB-50 psychiatric symptoms items.

A physical-psycho-social classification was used to identify the main reasons for consultation, resulting in different physical health and psychology variables, including social and professional aspects. This reflected the multiple impacts of COVID-19 in the individual's physical and psychological health and emergent worries due to socio-economical losses that reinforced the sense of loss and multiple grief/loss referred to as the secondary impact pandemic. Generically, during the quarter of confinement and self-confinement, new symptoms and unknown discomforts increased but still represented a minor number of consultations. Thus, the individuals receiving psychological support increased the existent symptoms, both in frequency and intensity, and those added by worries worsened in the current COVID-19 scenarios. Reports in Spain and several other countries also refer that the clinical history of patients worsened after the outbreak of the pandemic (5–7, 30). However, critical analysis by experts pointed out the resistance and resilience capacity of the general population being underestimated. They recommended careful administration of the limited resources to be able to respond to the needs of individuals already receiving psychological/psychiatric support and those identified as vulnerable or at risk “(28). In the present work, statistical analysis confirmed M+ diagnosis as a critical factor, with increases in most of the domains and items observed in M+ patients being significantly higher than those recorded in M- patients. This fact supported the design of the second part of the study, where M+ patients were explicitly assessed for their psychological/psychiatric burden with a clinical tool for this purpose.

In agreement with the nature of the sample and the medical psychology center, psychological aspects were the main topic of the consultations, with generalized fear been the most predominantly reported. The hypervigilance and hypersensitivity to stimuli, together with the need to carry out physical activities and live with “normality” in an atypical situation, was referred to by people with M+ as a real Molotov cocktail. A series of neighborhood conflicts occurred that would have been inconceivable for the participants. The third most consulted item referred to conflicts with neighbors. While scarcely reported before the pandemic, those neighbors' conflicts involving law enforcement and justice intervention strongly contributed to increased consultations. Neighbor relations were also the topic of analysis of social violence associated with COVID-19 in other countries (31).

While most literature on misophonia has focused on the clinical correlates and phenomenology of misophonia, some research works have also investigated its impact in work, school, social, and family domains (15, 32). In the present work, “family” was the second source of worries during the pandemic quarter since participants were highly concerned about relatives becoming infected and at risk of dying. Before the pandemic, participants were more concerned about the issues for which they were attending therapy, and the family was in a third position in the ranking of worries.

Physical health was the fourth reason for requests, followed by losing the job that already pointed at the economic crisis as a secondary pandemic resulting from COVID-19. Study-related consultations were not limited exclusively to patients who were part of regulated academic training. They also included consultations from participants who voluntarily attended training workshops related to misophonia organized by the medical center. These consultations referred to difficulties in performance, memory, attention, concentration, among other problems. Previous research in Chinese and American college student samples showed that regardless of the cultural contexts, the impairment may be significant for those with more frequent or severe misophonia levels than individuals with subclinical sound sensitivities (15, 32).

### Psychological and Psychosomatic Symptoms [PPS, LSB-50 Brief Symptom List)] in People With Misophonia

Regarding the prevalence of misophonia, research reports suggest that the number of people suffering misophonia is significant (15, 32). In the present work, the prevalence was 35%, being similar for women (37%) and men (31%). This is interesting to note since misophonia is quite unknown, leading to underdiagnosis and undertreatment. In a study that included a non-clinical sample of 483 undergraduate students, nearly 20% experienced clinically significant symptoms of misophonia (15). As indicated in different works, this syndrome can generate severe daily dysfunction (for example, occupational, interpersonal, academic), resulting in isolation, social, family, and couple conflicts. It can also contribute to the development of behavioral health problems (18), influence social life to extreme cases in which the individual may experience a decrease in mood or even have suicidal thoughts (10, 14, 18, 33)]. New preliminary studies also refer to the need to study misophonia and screen for comorbid psychiatric symptoms (34). In their work, PTSD (15.8%), OCD (11.5%), and MDD (9.6%) were found, in this order, the most common comorbidities. Similarly, the reports mentioned above on American and Chinese college students also provided evidence on misophonia symptoms being associated with substantial impairment and general sensory sensitivities, obsessive-compulsive, anxiety, and depressive symptoms (15, 32). Anxiety was found to significantly mediate the relationship between misophonia symptoms and anger attacks (32).

The symptom that had the highest percentage increase in women was “sleep disorder,” while in men it was “hostility” and “sleep disorder” was the second one ranked to increase severity. In both cases, women and men reported global insomnia (conciliation, maintenance, and early awakening), associated with the consequences of daytime activity such as irritability, fears, uncertainty, anxiety, depression/sorrow, worries about job loss, and family and neighbor tensions as a result of noise. At the same time, night-time reasons were recorded: excessive activity and noise from neighbors and noise from pets. These facts meant that people diagnosed with misophonia were in a hypervigilance state that led them to suffer from insomnia.

“Amplified sleep disorder” was the second most increased symptom, also with gender bias. This item is related to the manifestations of the anxiety and depression scales, symptoms which were found also increased. Similarly, patients reported a worsening in these areas. Even though sleep disturbances in the wake of traumatic events are well-known (35), the first clinical reports of COVID-19 revealing the immediate impact of the COVID-19 outbreak unveiled worrisome clinically significant insomnia, acute stress, anxiety, and depression, mostly in front-line workers but also on subjective sleep status of the general population (36–39). Public health emergency collections and task forces, such as the European CBTI Academy, provided practical recommendations to deal with sleep problems during confinement (40, 41). Thus, experts in the field recognize that the pandemic is causing a ‘second pandemic of insomnia’, and a new term Coronasomnia has been proposed.

The results of the present work on sleep and the fear to be infected, ill, or fear of dying are also relevant in the context of a recent review work that warns about the impact of the triad sleep insufficiency, anxiety, and psychosocial stress hampering immunity against viral infections and increasing the individual susceptibility to COVID-19 (42). According to these authors, the state-of-art of sleep, anxiety, and COVID-19 is still based on former research. Due to the emerging, and rapidly evolving situation of the COVID-19 pandemic, there is a strong need for further investigations as the virus seems meant to stay. Stress management measures, including addressing sleep-related disorders and sleep hygiene, are proposed to harness immune response and reduce viral infections' susceptibility (42).

After sleep disorders, hostility was the third variable to show a noticeable increase. It responds to the loss of emotional control with sudden or continuous aggression, anger, rage, or resentment. According to the results, an increase in these symptoms was observed in women and men. In both cases, patients justified these responses to the stress situation by confinement, excessive environmental and family noises, and, with particular relevance, children. The state of hypervigilance of day and night neighborhood behavior, changes in routines, fear, and uncertainty contributed to them. In this sense, it is important to note that while anger has been identified as the most prominent emotion in misophonia, irritation, stress and anxiety, aggravation, feeling trapped, and impatience have recently been reported as dominant emotions in some individuals (43).

Somatization was the fourth symptom that increased during the pandemic, twice as much in women than in men. Further evaluation showed that patients increased their physical and psychological distress during the pandemic, resulting in increased psychological and physical requests for therapist assistance.

The fifth variable was psychoactivity, with women increasing this symptom more than men. During the evaluation, patients recognized increased hypersensitivity to themselves and others. Especially and first of all, there is an increase in hypersensitivity to noise, followed by an increased sensitivity to lack of understanding in the couple and the extended family, to fears, worry, and uncertainty. This would be in agreement with recent reports about intolerance to uncertainty, which seems to be underlying the symptoms of anxiety and depression disorders (44), and to loneliness found during this pandemic, mostly in older adults (45, 46).

The following symptom that increased most during the pandemic was anxiety, more in women than in men. According to subsequent analysis, 12 patients reported having a panic attack during the pandemic: 6 with emergency room care, 1 with hospital admission. They also referred to increased fears in general, especially the contagious situation, both in themselves and their families. Half of the patients reported symptoms of anxiety-related to noise from family, pets, and neighbors.

Depression was the seventh variable with increased symptoms, more in women than in men. According to the clinical interview, they accounted for the increase during the pandemic quarter due, first, to the situation of uncertainty, lack of rest, loneliness, difficulty in adapting to the sudden change brought about by the pandemic, feelings of guilt and helplessness, emotional state, and irritability of both them and their family members.

The eighth item was obsession-compulsion. Patients scoring for this variable reported an increase in self-conversation. They referred to the behaviors they would have with neighbors and family members if they would make certain noises, in the same way as they do with those derived from work problems and conflicts. They also reported negative thoughts about their health and that of their family members. They acknowledged in the same way, having increased normal and some superstitious rituals. Several reports on the impact of COVID-19 on mental health point out the increase of anxiety-related symptoms and the current scenarios as a trigger on obsessive-compulsive disorder (47–51).

Finally, both women and men generically recognize that they feel particularly vulnerable to events arising from the pandemic situation, as reflected by hypersensitivity.

## Conclusions and Future Perspectives

The present study provides evidence that the period of confinement and self-confinement enhanced the impact of misophonia in daily life activities and the well-being of people with severe aversive responses to certain sounds and movements. The data confirmed the strong capacity of misophonia to disrupt the participants' mental health in this unprecedented confinement context. Although the present work did not assess the quality of life (QoL) with a tool, a considerable decrease was predicted from the worsening of PPS, as also referred by participants, deserving future endeavors. The present work also shows that stressors were associated with the pandemic situation in general and, in particular, with the containment and self-confinement situation. Health, fears, conflicts with neighbors, study-related difficulties were outstanding reasons for consultations. Psychological and Psychosomatic Symptoms in participants diagnosed with misophonia revealed an increase of all items. Sleep disorders (coronasomnia), hostility, depression, and somatization were the most severe than previous assessments. From a gender perspective, in most of the variables, women presented the worst psychological and psychosomatic states that demanded more substantial therapeutical efforts.

There was a change in behaviors concerning the therapeutic intervention: most patients who felt more emotionally secure postponed their therapeutic schedules to later. Patients with misophonia kept their schedules for two reasons, (1) fear of not being able to address the problem, especially that arising from the excessive noise they anticipated during confinement, and (2) because they were aware of the lack of therapeutic and professional support regarding the problem of misophonia outside the therapeutic setting they were carrying out.

This study reveals that besides the pandemic's effects on the general population, patients diagnosed with misophonia suffered an increase in symptoms due to the trigger sounds associated with neighborhood and family members. Lack of family, neighborhood, and professional understanding, due to lack of knowledge of misophonia, exacerbated psychological and psychosomatic problems during the pandemic.

Another aspect of great importance was the increase in interpersonal conflicts; within families and neighbors' communities. Since post-traumatic stress disorder has been related to the severity of the misophonic symptoms (34), the post-COVID scenario's perspectives are complex in this respect. Our results can help develop coping strategies addressing modifiable risk and protective factors for each mental status for early implementation in future outbreaks.

In summary, the study unveiled the complex physical-psychological-social burden, the need for dissemination, and a gender perspective to understand and approach the vast array of secondary impacts due to the COVID-19 pandemic on the population with misophonia. The unfeasibility to implement one-model-fits-all also highlights the relevance of further research investigation related to misophonia in the current and post-COVID-19 scenarios.

## Data Availability Statement

The raw data supporting the conclusions of this article will be made available by the authors, without undue reservation.

## Ethics Statement

The studies involving human participants were reviewed and approved by L'Alfatier. The patients/participants provided their written informed consent to participate in this study.

## Author Contributions

AF-T: interviews, data recordings and analysis, and manuscript draft. AF-T and LG-L: equally contributed to the concept, design, and scientific discussion, and the writing and approval of the manuscript. All authors have equally contributed to the article and approved the submitted version.

## Conflict of Interest

The authors declare that the research was conducted in the absence of any commercial or financial relationships that could be construed as a potential conflict of interest.
